# Fistula first, catheter last: can the mouth be second?

**DOI:** 10.3389/fneph.2024.1385544

**Published:** 2024-05-23

**Authors:** Karo Parsegian, Jonathan Himmelfarb, George Fares, Effie Ioannidou

**Affiliations:** ^1^ Division of Periodontics, Department of Diagnostic Sciences and Surgical Dentistry, School of Dental Medicine, University of Colorado Anschutz Medical Campus, Aurora, CO, United States; ^2^ Center for Kidney Disease Innovation Icahn School of Medicine at Mount Sinai, New York, NY, United States; ^3^ Baystate Medical Center, Springfield, MA, United States; ^4^ Department of Orofacial Sciences, School of Dentistry, University of California, San Francisco, San Francisco, CA, United States

**Keywords:** CKD/ESRD, fistula, inflammation, periodontal therapy, interdisciplinary treatment approach

## 1 Introduction

Chronic kidney disease (CKD) is characterized by persistent alterations in kidney structure and impaired excretory renal function and represents a public health burden affecting ~14.5% of the U.S. adult population (1). Even at early disease stages, increased urea concentration is associated with elevated serum levels of various pro-inflammatory mediators, including high-sensitivity C-reactive protein (hs-CRP), interleukin-6 (IL-6), and tumor necrosis factor-alpha (TNFα) (1–4). These changes become even more pronounced as CKD progresses to end-stage renal disease, ESRD (2, 3). Given the importance of pro-inflammatory biomarkers as predictors of all-cause (5) and cardiovascular disease (CVD) (6) mortality in patients with ESRD, reductions in their levels have been proposed as critical target outcomes in this population (3, 4). Several anti-inflammatory strategies, including pharmacological (4) and non-pharmacological (mostly nutritional) (3, 4, 7, 8) interventions and the concurrent therapy of systemic comorbidities (9), have been utilized in that direction.

Hemodialysis (HD) is the primary treatment modality in ESRD. The most recent national data demonstrated that in >70% of patients with ESRD, HD was initiated with a central venous catheter (CVC) (10), which could later be replaced with an arteriovenous fistula (AVF) or arteriovenous graft (AVG) (11). Although AVF was associated with various complications, it provided the lowest rate of mortality, fatal infections, and levels of pro-inflammatory mediators compared to AVG and CVC (11–13). Even in the absence of infection, patients with CVC had significantly higher serum hs-CRP levels than those with AVF independent of sex, race, and diabetes mellitus (DM) status (12). Conversely, the CVC-to-AVF switch significantly reduced serum levels of pro-inflammatory mediators (12) and all-cause mortality rates (11). The accumulating evidence on the CVC-to-AVF switch to optimize the HD outcomes led to the development of the national ESRD Network Initiative called “Fistula First”, sometimes referred to as “Fistula First, Catheter Last” (14).

Peritoneal dialysis (PD) is another treatment modality in ESRD that offers comparable patient survival outcomes, reduced risk of septicemia, improved health-related life quality, and a more flexible lifestyle than HD. Consequently, other countries have developed the “PD first” initiative for access to care and affordability reasons, which increased the number of patients receiving PD (15). In the U.S., the 2019 Advancing American Kidney Health Executive Order contributed to the increased utilization of PD (16), and the developed North American PD Catheter Registry has offered an extensive dataset of PD outcomes (17).

Kidney transplant (KT) from living and deceased donors is another kidney replacement modality, which in the absence of contraindications offers an increased survival rate (18) and life quality (19) compared to HD. Paradoxically, oral health becomes relevant only at the pre-transplant stages, when clearance is required to proceed with the transplant process, while ignored during CKD/ESRD stages.

## 2 Oral cavity as an additional source of inflammation in patients with CKD/ESRD

Oral tissues are continuously exposed to ~800 bacterial species (20), and the chronic inflammatory infiltrate is present even in clinically healthy tissues (21). Therefore, it is critical to recognize that the oral cavity, an important modifiable source of inflammation in patients with ESRD, is frequently overlooked (22).

Among oral inflammatory conditions, this perspective focused on periodontitis, a polymicrobial multifactorial inflammatory disease of tooth-supporting tissues that affects 42% of the U.S. adult population (23). The Global Burden of Disease study confirmed the burden of periodontitis as the sixth most prevalent disease, with an estimated 54 billion USD/year cost of productivity loss worldwide (24–26). Once developed, it is characterized by the progressive, life-long destruction of connective tissue and alveolar bone surrounding teeth, which often leads to tooth loss (27).

## 3 The conceptual model of the increased prevalence of periodontitis in patients with CKD/ESRD

**Figure 1:**
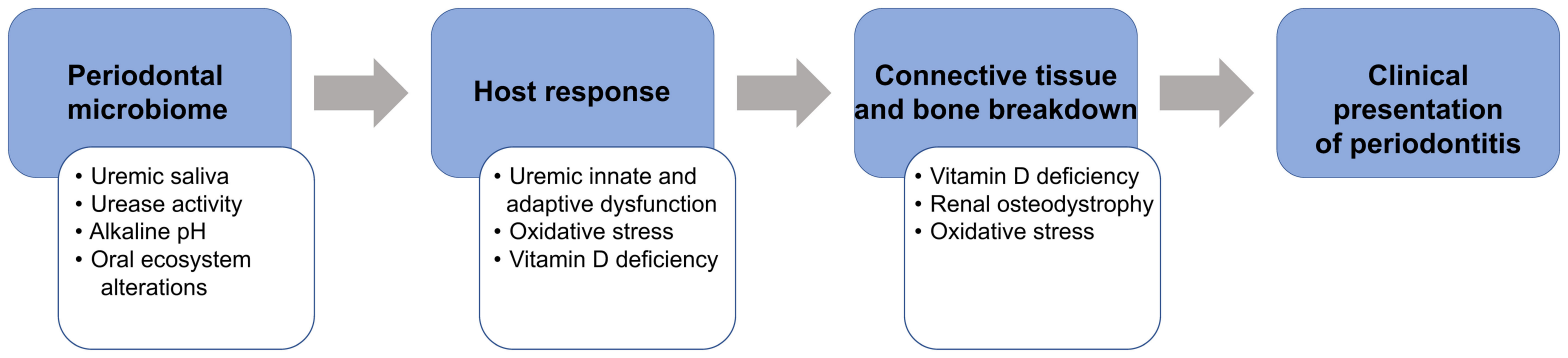
The proposed conceptual model that explains the modifying ESRD role (white boxes) on the pathogenesis of periodontitis (blue boxes).

Using the National Health and Nutrition Examination Survey III database, we demonstrated significantly higher periodontitis prevalence in patients with CKD compared to the general population accentuated by racial disparities in CKD (35.28% (28) vs. 7.8% (29), respectively). Despite the recognition of periodontitis as a critical public health problem (30, 31), its awareness in CKD/ESRD populations remains low (32). Therefore, oral health promotion, effective treatment modalities, and disease prevention focused on reducing oral and systemic inflammation and oxidative stress should be prioritized in these populations. Our conceptual model describes the underlying mechanisms of the increased prevalence of periodontitis in patients with CKD/ESRD, assesses the contribution of periodontitis to the systemic inflammatory burden, and determines the extent of anti-inflammatory effects of standard-of-care non-surgical periodontal therapy (NSPT) in these patients (**Figure 1**).

### 3.1 Biologic plausibility for the increased prevalence of periodontitis in patients with CKD/ESRD

Periodontitis involves the interaction of periodontal pathogens in subgingival dental biofilm with the susceptible host and environmental factors, resulting in destructive changes within the connective tissue and alveolar bone. In CKD/ESRD, several mechanisms, including uremic effects on the oral environment and host immune response and behavioral factors (33, 34), can contribute to the pathogenesis of periodontitis. With the progressive loss of glomerular filtration rate (GFR), uremic solutes/toxins, normally eliminated with the urine, are retained in the body (35), contributing to anemia, immunosuppression, inflammation, infection, CVD morbidity, and mortality. Deleterious effects of increased urea toxins are mostly pronounced in ESRD (36). The proposed conceptual model, which explains the modifying role of ESRD (**Figure 1**, white boxes) on the pathogenesis of periodontitis (**Figure 1**, blue boxes), is summarized below. As urea concentration becomes pronounced (uremia), salivary urea levels dramatically increase from 7.5 to 17-26 μmol/L (37), altering the oral ecosystem and promoting the shift from microbial symbiosis to dysbiosis (38–40), similar to the uremia-related changes observed in the gut environment (41). Additional effects of the uremic milieu on innate and adaptive immunity (42) alter host response and increase infection susceptibility, as confirmed by a high prevalence of chronic infection associated with *Chlamydia pneumoniae* and *Mycobacterium tuberculosis* (43), the high relative risk for tuberculosis (44, 45), and the high prevalence of periodontitis. Also, vitamin D deficiency plays a dual role in abnormal bone breakdown/turnover (renal osteodystrophy) (46) and alteration of both innate (monocyte activation and phagocytosis) and adaptive immunity (modulation of cytokine production) (47), which could further promote periodontal tissue breakdown.

### 3.2 Periodontitis and the inflammatory burden in patients with CKD/ESRD

Within the last decades, the high inflammatory burden in ESRD was attributed to the “uremic puzzle.” The pieces included risk factors related to inflammation, endothelial function, vascular ossification markers, and uremic CVD markers, developing and connecting intricately (48), and contributing to CVD mortality. We now know that the complexity of the “uremic puzzle” extends past the Framingham CVD risk factors involving systemic inflammation and oxidative stress as the variables strongly associated with poor CVD outcomes in CKD (48). Overwhelming evidence indicated that the enzymatic myeloperoxidase activity linked oxidative stress and inflammation in uremia, emphasizing the importance of both events in the atherosclerotic process (49, 50). Consequently, research became focused on identifying dialysis-related factors (such as membrane bio-incompatibility, type of access, and impure dialysate) that contribute to systemic inflammation and oxidative stress at various levels (51). At the patient level, additional comorbid factors, including bacterial infections, volume overload, inadequate dialysis, and depression, were implicated in the elevation of hs-CRP and targeted in anti-inflammatory therapeutic efforts (52).

In the same context, the association between periodontitis and systemic inflammation was evidenced by higher levels of pro-inflammatory mediators in patients with periodontitis. Using a parsimonious regression model, our group previously showed that periodontitis led to the increased extent of inflammation in patients with CKD, as evidenced by an ~100% increase in the odds of elevated serum hs-CRP levels (odds ratio, OR, 2.0, 95% confidence interval, CI, 1.2, 3.6; *P* = 0.02) (53). Moreover, the impact of periodontitis on serum hs-CRP levels in patients with ESRD significantly correlated with the periodontitis severity (2.4, 4.2, and 4.4 mg/L in the slight, moderate, and severe periodontal tissue breakdown, respectively) (54). Recent evidence suggested that periodontitis could contribute to renal structural alterations (55), possibly via increased local and systemic oxidative stress and a compromised antioxidant capacity (56). However, precise mechanisms linking these events remain to be explored further.

As confirmed by analogy (57), bacterial infections increased levels of inflammatory mediators in patients undergoing HD, thus contributing to increased mortality (58, 59). Therefore, the CKD/ESRD - periodontitis relationship might be bi-directional in a way that CKD/ESRD potentiates the incidence of periodontitis, and periodontitis contributes to the sustained inflammation and poor outcomes in CKD/ESRD. This is indirectly confirmed by the meta-analysis of seventeen studies that showed a higher risk of developing CKD in patients with periodontitis than those without periodontitis (OR 1.60; 95% CI 1.44, 1.79; *P* = 0.11) (60). At the same time, patients with CKD had a similarly higher risk of developing periodontitis compared to control patients without CKD (OR 1.69; 95% CI 0.84, 3.40; *P* < 0.001) (60).

### 3.3 Oral and systemic effects of NSPT

NSPT involves the mechanical removal of dental biofilm (the microbial etiology) and calculus deposits (the main predisposing factor) from supra- and subgingival tooth surfaces (27). The treatment response is based on evaluating clinical periodontal determinants 4-6 weeks post-NSPT, and the disease progression is controlled through regular periodontal maintenance visits (typically every three months) (27). Systemic anti-inflammatory effects of NSPT first require a positive treatment response at the local/oral level with improved periodontal measures (61). In both systemically healthy and ESRD populations, NSPT improved periodontal clinical parameters and levels of systemic inflammatory markers (62).

Only short-term (<6-month follow-up) randomized controlled trials (RCTs) have examined the effects of NSPT on hs-CRP levels in patients undergoing HD and/or PD. The meta-analysis of these studies showed that NSPT significantly but moderately decreased serum hs-CRP levels (standardized mean difference, SMD, -1.53; 95% CI -2.95, -0.11; *P* < 0.001) (63). This reduction in hs-CRP following the NSPT appeared to be of a similar magnitude to the hs-CRP reduction observed following the CVC-to-AVF switch (OR 1.43; 95% CI 1.15, 1.68; *P* ≤ 0.05) (58). The levels of other pro-inflammatory mediators (IL-6 and TNFα) were not affected by NSPT (SMD, -0.23; 95% CI -0.78, 0.33; *P* > 0.05), even when examined at later time points (63).

It is important to highlight several key limitations in the current evidence of the matter. *First*, a similar decrease in hs-CRP protein concentration does not necessarily translate into clinical effects. Therefore, rigorous studies comparing the effects of NSPT and the CVC-to-AVF switch are needed. *Second*, as mentioned above, the systemic anti-inflammatory effect of NSPT requires a positive treatment response at the local/oral level with improved periodontal measures. Therefore, the efficacy of NSPT in trials that do not achieve clinically acceptable periodontal endpoints before evaluating systemic endpoints should be questioned. *Third*, as NSPT requires periodontal maintenance to minimize periodontal tissue breakdown, the episodic treatment approach with erratic maintenance visits is ineffective enough to promote optimal oral hygiene, prevent reactivation of periodontitis, and control systemic inflammation. *Fourth*, only short-term RCTs examined the effects of NSPT on the levels of systemic pro-inflammatory markers, which does not allow for assessing the sustainable anti-inflammatory therapeutic effects. *Fifth*, the discrepancies in the prevalence of systemic inflammatory conditions (such as DM) in the treatment vs. control arms and a well-documented association between periodontitis and DM make interpreting the results challenging. *Finally*, study design limitations include a moderate overall risk of bias (defined by low or unclear allocation concealment, blinding of participants, personnel, and outcomes) and lack of essential information about the included cohorts (such as the dialysis duration).

## 4 Discussion

In the present work, we proposed the conceptual model of the association between periodontitis and CKD/ESRD and provided evidence that outcomes of NSPT could be as effective in reducing inflammatory burden in patients with CKD/ESRD as the CVC-to-AVF switch. We argue that effective anti-inflammatory strategies in patients with CKD/ESRD should include multidisciplinary collaboration, oral health promotion, effective treatment strategies, and disease prevention focused on reducing oral and systemic inflammation. In this regard, outpatient dialysis centers could serve as an archetype for continuous in-center oral health maintenance care delivery. Future research is needed to examine systematic and repeated oral health delivery models in dialysis units to improve the periodontal status and modulate systemic inflammation in patients with ESRD.

## Author contributions

KP: Writing – original draft, Writing – review & editing. JH: Conceptualization, Writing – review & editing, Writing – original draft. GF: Writing – review & editing. EI: Conceptualization, Funding acquisition, Methodology, Writing – original draft, Writing – review & editing.
